# How to Easily Make Self-Sensing Pneumatic Inverse Artificial Muscles

**DOI:** 10.3390/biomimetics9030177

**Published:** 2024-03-15

**Authors:** Valentina Potnik, Gabriele Frediani, Federico Carpi

**Affiliations:** 1 Biomedical Engineering Unit, Department of Industrial Engineering, University of Florence, 50121 Florence, Italy; valentina.potnik@unifi.it (V.P.); frediani.gabriele@gmail.com (G.F.); 2 IRCCS Fondazione don Carlo Gnocchi ONLUS, 50143 Florence, Italy

**Keywords:** actuator, pneumatic, self-sensing, artificial muscle

## Abstract

Wearable mechatronics for powered orthoses, exoskeletons and prostheses require improved soft actuation systems acting as ‘artificial muscles’ that are capable of large strains, high stresses, fast response and self-sensing and that show electrically safe operation, low specific weight and large compliance. Among the diversity of soft actuation technologies under investigation, pneumatic devices have been the focus, during the last couple of decades, of renewed interest as an intrinsically soft artificial muscle technology, due to technological advances stimulated by applications in soft robotics. As of today, quite a few solutions are available to endow a pneumatic soft device with linear actuation and self-sensing ability, while also easily achieving these features with off-the-shelf materials and low-cost fabrication processes. Here, we describe a simple process to make self-sensing pneumatic actuators, which may be used as ‘inverse artificial muscles’, as, upon pressurisation, they elongate instead of contracting. They are made of an elastomeric tube surrounded by a plastic coil, which constrains radial expansions. As a novelty relative to the state of the art, the self-sensing ability was obtained with a piezoresistive stretch sensor shaped as a conductive elastomeric body along the tube’s central axis. Moreover, we detail, also by means of video clips, a step-by-step manufacturing process, which uses off-the-shelf materials and simple procedures, so as to facilitate reproducibility.

## 1. Introduction

Several fields of application currently in rapid expansion require improved soft actuation systems acting as ‘artificial muscles’ that are capable of large strains, high stresses, fast response and self-sensing and that show electrically safe operation, low specific weight and large compliance. As an example, one of those fields is wearable mechatronics, which aims to tightly interface the human body with actuation devices; such devices can find application in the creation of powered versions of systems as different as orthoses (to assist people with disabilities), exoskeletons (to augment body movements and forces) and prostheses (to replace missing body parts) [1,2,3,4,5]. Specific uses of such systems may benefit from actuators that have a soft structure, especially to increase the systems’ wearability and acceptability by users.

The state of the art of soft actuators includes a diversity of technologies, which can be classified into two main groups: (i) actuators with intrinsically soft structures, such as fluidic devices [6] and electromechanically active polymers [7,8,9,10,11,12,13]; (ii) actuators with extrinsically soft structures, such as elastomeric bodies that are deformed by embedded motor-pulled cables [14,15,16] or by embedded thermally shrinking fibres; the latter can be made, for instance, of shape memory alloys [17] or twisted-and-coiled polymers [18]. Furthermore, temperature-activated materials can also be used without any soft structure to create flexible linear artificial muscles that are just made of SMA fibres [19] or twisted-and-coiled polymer fibres [20].

Though each technology has its pros and cons, the field of artificial muscle actuators for wearable mechatronics is dominated by pneumatic devices, especially when a miniaturisation of the actuator, including its driving unit, is not important. Indeed, all the requirements stated above can be met by soft pneumatic actuators [21,22], although it should be kept in mind that a high performance is usually paired with the need for relatively bulky (and noisy) electropneumatic units. Therefore, it should not be surprising that pneumatic devices have been the focus, during the last couple of decades, of renewed interest as an intrinsically soft artificial muscle technology. Furthermore, they benefit from technological advances that are continuously stimulated by a growing number of applications in soft robotics [23].

The most common example of soft pneumatic actuators is represented by the McKibben configuration, consisting of a gastight elastomeric bladder surrounded by a braided net, which transduces radial expansions into axial contractions when the bladder is pressurised; these types of soft contractile actuators (and similar alternatives) are usually referred to as ‘artificial muscles’, because of their functional similarity with skeletal muscles [24,25,26]. Some McKibben actuators have also been provided with self-sensing ability, by using various strategies, such as piezoresistive sensors consisting of helical microchannels filled with eutectic gallium–indium [27] or piezoinductive sensors made of braided electrical wires [26].

Soft pneumatic actuators can also be designed in such a way that, upon pressurisation, they elongate instead of contracting; in this case, they are often referred to as ‘inverse artificial muscles’. An efficient configuration to achieve this effect is represented by an elastomeric inflatable tube surrounded by a plastic coil, which restricts radial expansions upon inflation, enabling only elongations [28,29]. Planar and textile-compatible versions of this concept have also been demonstrated, by radially constraining fluidically driven small elastomeric tubes via a surrounding fabric, which can integrate the tubes in various ways [30,31,32]. The coil-based versions are particularly attractive for applications at various scales, as linear motions with significant performance can be achieved using a simple architecture, which keeps their lateral size limited and simplifies manufacturing.

Here, we present a preliminary investigation aimed at endowing coil-based inverse artificial muscles with self-sensing ability, while maintaining ease of fabrication, especially with off-the-shelf materials and low-cost processes. To the best of our knowledge, so far only one paper has presented a self-sensing version of coil-based inverse artificial muscles: a piezoresistive sensor was created by making the coil as an insulated elastomeric conductive cord, such that it had not only a mechanical function (constraining of radial strains) but also an electrical function [33].

**Figure 1:**
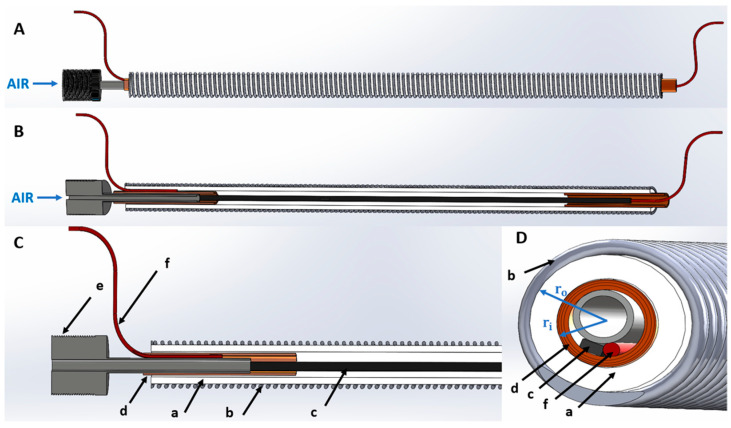
Drawings of the proposed self-sensing pneumatic inverse artificial muscle: (**A**) lateral view; (**B**) sectional view; (**C**) magnified sectional view of the left-hand end, showing the different parts of the structure: (a) elastomeric tube, (b) plastic coil, (c) piezoresistive stretch sensor, (d) copper tape for electrical contacts, (e) air needle, (f) electrical wire for resistance reading; (**D**) cross-sectional view.

In this paper, we describe an alternative strategy for piezoresistive self-sensing, achieved by shaping a piezoresistive stretch sensor as a conductive elastomeric linear body arranged along the tube’s central axis. A schematic of the whole device is shown in Figure 1.

This solution shows some useful features: (i) it exploits the empty internal space of the actuator; (ii) it does not require any modification to the constraining plastic coil, thereby not only simplifying manufacturing, but also especially maintaining its optimal functionality; indeed, the coil should be flexible but not stretchable, so as to restrain radial expansions, which otherwise would decrease the actuator’s axial output mechanical work; (iii) it makes the sensor sensible only to axial elongations and lateral bending of the actuator, but not also to radial expansions (which otherwise may be possible with an elastomeric sensing coil); this way, the sensitivity to axial strains is expected to be higher.

In the following, we detail, also by means of video clips, a step-by-step manufacturing process, which uses off-the-shelf materials and simple procedures, so as to facilitate reproducibility.

## 2. Materials and Methods

### 2.1. Elastomeric Tube

The actuator can be manufactured with any soft, airtight elastomeric tube. As a constitutive material, we opted for a silicone elastomer. A tube with a hardness as low as 40 ShoreA was purchased from LegenDay Technology Co., Shenzhen, China. The tube had inner and outer radii *r_i_* = 1.5 and *r_o_* = 2.5 mm, respectively (Figure 1D). In order to fabricate actuators with a final active length of 100 mm, samples with a length of 110 mm were cut from the tube, in consideration of the need for creating mechanical and electrical connections at the extremities.

### 2.2. Elastomeric Piezoresistive Sensor

The conductive elastomeric linear body to be used as an axial piezoresistive sensor could be made of any deformable conducting material. We opted for carbon-loaded silicone elastomers, as they typically are a well-established solution to obtain high-quality piezoresistive stretchable sensors. Several types of conductive soft cords with circular a cross section are available on the market. An example is represented by cords manufactured by Euro Technologies, Concorezzo (MB), Italy [34]. In this investigation, for simplicity, we used a thin planar stripe obtained by cutting a commercial conductive rubber sheet (5464, Adafruit, New York, NY, USA) already available in our lab. The stripe had a thickness of 0.5 mm, a width of 2 mm and a length of 106 mm.

In order to fabricate electrical contacts to read the resistance, each extremity of the stripe was joined to an electrical wire, and their junction was stabilised by tightly wrapping a piece of a flexible copper tape around them.

Prior to fabricating the self-sensing actuator, the quasi-static sensing properties of the piezoresistive stripe were verified, by stretching it and measuring the variation of its electrical resistance.

### 2.3. Fabrication of the Self-Sensing Pneumatic Inverse Artificial Muscle

#### 2.3.1. Step 1: Preparation of the Restriction Coil

**Figure 2:**
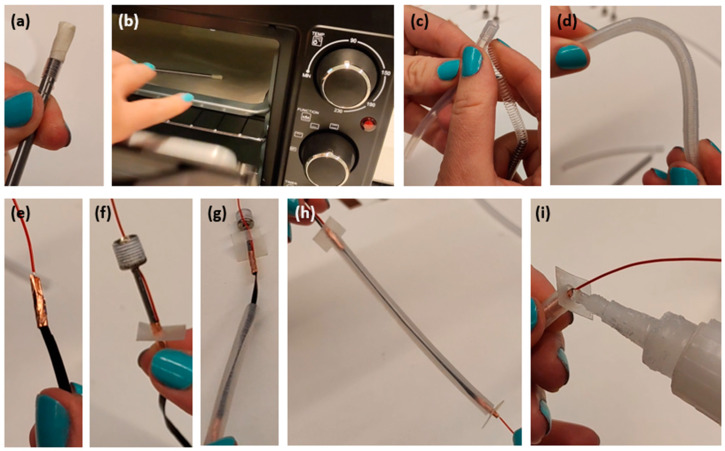
Sequential phases of the fabrication of the self-sensing pneumatic inverse artificial muscle: (**a**) coiling of a Nylon filament around a metal rod; (**b**) heating in an oven of the coil wrapped around the rod, for thermal annealing; (**c**) recoiling of the annealed Nylon coil around the silicone tube; (**d**) verification of the coil’s adherence to the tube upon bending; (**e**) creation of an electrical connection between an extremity of the elastomeric sensor and an electrical wire, by wrapping copper tape around them; (**f**) application of an air needle to one of the two extremities of the sensor; (**g**) insertion of the sensor into the actuator; (**h**) application of a plastic sheet to each end of the structure; (**i**) final glueing.

The plastic coil to be arranged around the elastomeric tube (in order to restrict its radial expansion upon pressurisation) consisted of a Nylon filament (fishing line), having a diameter of 0.5 mm. The filament was coiled around a stiff (metal) rod (Figure 2a), which had a diameter equal to the silicone tube’s inner diameter. The coil on the rod was subjected to thermal annealing, consisting of heating in an oven at 90 °C for 20 min (Figure 2b), followed by ambient cooling. As a result, the Nylon filament acquired a stable coiled shape.

This phase of the process is documented in the Supplementary Video ‘Fabrication step 1’.

#### 2.3.2. Step 2: Application of the Restriction Coil

The annealed Nylon coil was removed from the rod and recoiled around the silicone tube, making sure that it tightly adhered to it (Figure 2c). As a result, the coil was able to maintain a close fit around the tube, even when the structure was subjected to bending (Figure 2d). It is worth noting that recoiling was found to be necessary, as attempts to directly transfer (by sliding) the coil from the rod to the tube did not ensure proper adherence.

This phase of the process is documented in the Supplementary Video ‘Fabrication step 2’.

#### 2.3.3. Step 3: Creation of the Sensor and Its Electrical Contacts

A piezoresistive sensing stripe was created as described in Section 2.2. Its electrical contacts were fabricated by joining each end of the stripe to an electrical wire and stabilising their junction by tightly wrapping a flexible copper tape around them (Figure 2e). At one of the two ends of the actuator, the junction between the sensor’s extremity and electrical wire also included an air needle (Figure 1D and Figure 2f), which was used to pneumatically connect the actuator to a tubing from the driving compressor. The various parts were bonded together using an adhesive glue (Loctite 495, Henkel, Düsseldorf, Germany).

This phase of the process is documented in the Supplementary Video ‘Fabrication step 3’.

#### 2.3.4. Step 4: Combination of the Actuator and Sensor and Sealing of the Structure

The sensor was inserted into the actuator (Figure 2g), obtaining an integrated device (Figure 2h). In order to make the structure airtight and create a mechanical interface to apply external loads, each extremity was closed with a piece of a plastic sheet and sealed (Figure 2i) using the same Loctite glue employed during the preceding step. The glue ensured adequate bonding between the silicone tube and the plastic sheet. It is worth noting that sealing may not be achievable with any type of glue, as silicone is known to be difficult to bond to different materials.

This phase of the process is documented in the Supplementary Video ‘Fabrication step 4’.

**Figure 3:**
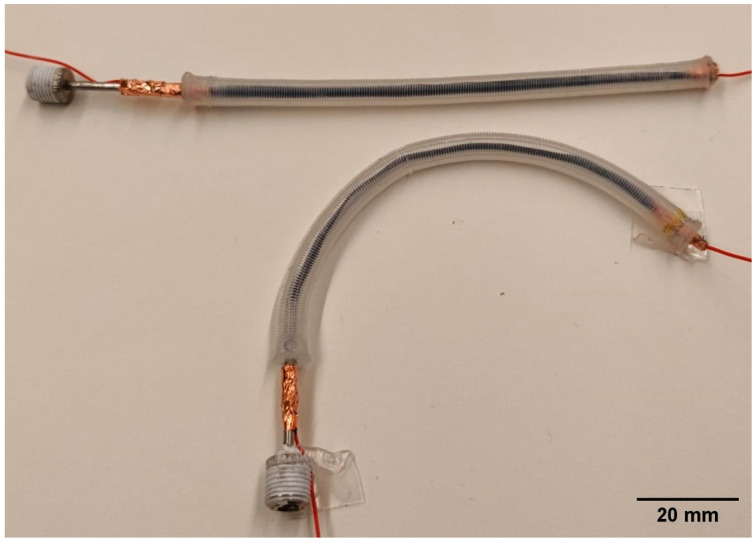
Prototype samples of the self-sensing inverse artificial muscle actuator.

Figure 3 shows two samples of the assembled self-sensing actuator, which had a natural free length of ~100 mm.

### 2.4. Measurement of the Actuation Free Stroke

The quasi-static axial elongation in response to stepwise pressure signals with variable amplitude was measured on samples vertically arranged in isotonic condition, which was established by applying a constant load with a hanging mass. These free stroke measurements were performed at different loads: 0, 100 g, 200 g, 300 g, 400 g and 500 g.

In order to calculate the prestresses corresponding to these loads, the relevant total cross-sectional area was calculated as the sum of the following two contributions: (i) the cross-sectional area of the sensor; (ii) the cross-sectional area of the tube’s wall, i.e., the annular area identified by the tube’s wall thickness *r_o_−r_i_* (Figure 1D). Accordingly, the loads corresponded to axial prestresses of about 0, 72, 145, 217, 289 and 361 kPa. It is worth noting that, for the sake of these calculations, the coil’s cross section was excluded, as the effect of any applied load on the coil mostly consisted of just a change in pitch.

The elongations were measured for both increasing and decreasing values of pressures, up to 3 bars and down to 0, so as to investigate the actuator’s hysteretic behaviour.

Prior to starting the measurements with any new load (applied mass), the sample was preloaded with that mass for 10 min so as to wait for a stabilisation of its lengthening due to viscoelastic creep. Then, the pressure was changed in steps of 0.2 bars (starting from 0.4 bars).

This characterisation was repeated on three samples.

### 2.5. Measurement of the Actuation Blocking Force

The quasi-static axial force generated in response to stepwise pressure signals with variable amplitude was measured with a load cell on samples vertically arranged in isometric condition. The samples were tested at their natural length, i.e., without any prestretch. The pressure was increased in steps of 0.5 bars, up to 3 bars.

The isometric condition necessary for this blocking force test was implemented as follows. Vertical clamping alone was found to be insufficient to enable proper operation. Indeed, as the actuator was much longer than wide, pressurisations while it was clamped at its natural length tended to cause lateral instabilities. Therefore, the actuator was constrained not only vertically but also radially, by inserting it into a stiff, hollow cylinder, whose inner diameter was slightly bigger than the actuator’s diameter (their surfaces were separated by a 0.5 mm gap).

This characterisation was repeated on three samples.

### 2.6. Measurement of the Actuator’s Self-Sensing Performance

The quasi-static variation of the internal sensor’s electrical resistance due to stepwise pressure signals with variable amplitude was continuously monitored with a multimeter during the isotonic actuation tests. As a result, the test returned a set of resistance values corresponding to pressurisations and depressurisations at the different applied loads (0, 100 g, 200 g, 300 g, 400 g and 500 g).

In order to identify the constitutive relationship between the sensor’s output and the actuator’s strain, the relative variation of the resistance was also plotted as a function of the strain.

This characterisation of the sensor’s performance was repeated on three samples.

## 3. Results and Discussion

**Figure 4:**
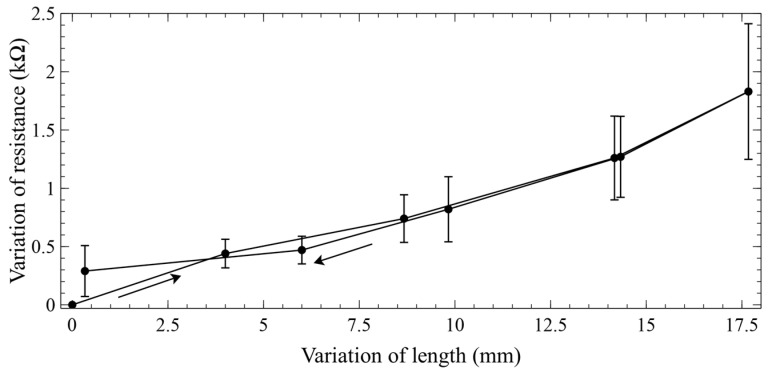
Verification of the quasi-static sensing properties of the piezoresistive stripe, prior to its integration with the actuator: variation of its resistance in response to increasing and decreasing elongations (indicated by arrows). The stripe had a prestrain of 12%. The error bars represent the standard deviation among three samples. Linear interpolations of the data are used as a guide for the eye.

Figure 4 presents a verification of the sensing properties of the piezoresistive stripe (prior to its integration with the actuator), which was subjected to one cycle of stretching and releasing (by hanging it and loading and unloading it with variable applied weights).

**Figure 5:**
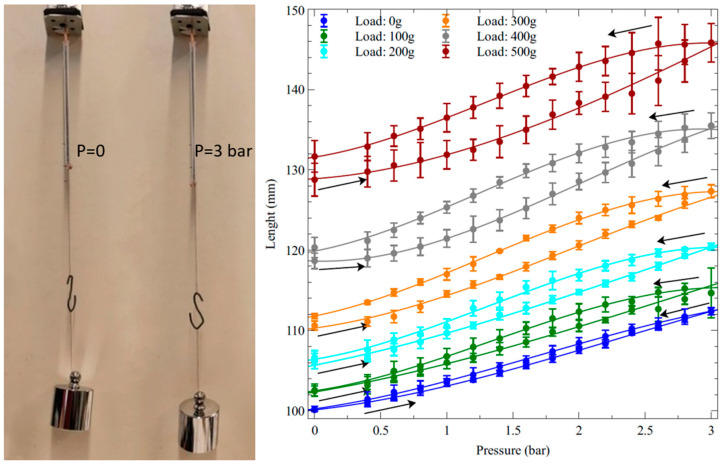
Elongations of the inverse artificial muscle actuator in response to the applied pressure at different loads. (**Left**): photos of the experimental setup, showing the same actuator at a pressure of 0 and 3 bars, with a hanging mass of 500 g. (**Right**): plots of the actuator length as a function of pressurisations and depressurisations (indicated by arrows). The error bars represent the standard deviation among three samples. Fitting curves are used as a guide for the eye.

This test on the stripe was performed with a prestrain relevant to its envisaged use inside the actuator. In particular, prior to starting the test, the stripe was prestrained by approximately the same amount experienced within the actuator, when the latter was tested with the applied load (300 g, as reported in the following) that caused the highest pressure-induced strains (~15% at 3 bars, as reported in the following). That condition for the actuator corresponded to a prestrain of ~11% at a null pressure (as is visible in Figure 5). Consistently, the sensing stripe was preloaded to reach a comparable prestrain (~12%); then, during the test, it was progressively further stretched by applying increasing loads, reaching a maximum elongation of ~17.7 mm (Figure 4), corresponding to a maximum strain of 16% (from the prestrained state).

A characterisation of the active elongations produced by pressurisations and depressurisations of the self-sensing inverse artificial muscle actuator at the different loads is presented in Figure 5.

The left-hand side of the figure shows the experimental setup and an actuator in action, which was imaged, for comparison, at zero pressure and the maximum pressure tested. The right-hand side of the figure illustrates how the elongation varied as a function of the applied pressure, at different loads, during the pressurisation and depressurisation phases.

As visible from the photos in Figure 5, the pressurisations did not cause any lateral bulging of the tube (at least up the maximum pressure of 3 bars tested in this study). This means that, under pressurisation, the coil maintained proper adherence to the tube throughout its elongation. This important feature was enabled by the thermal annealing of the Nylon filament, which provided the coil with a stable geometrical configuration. An analysis of the literature showed that an analogous beneficial effect has previously been reported also for fluidically driven artificial muscle actuators with stainless steel coils [35].

**Figure 6:**
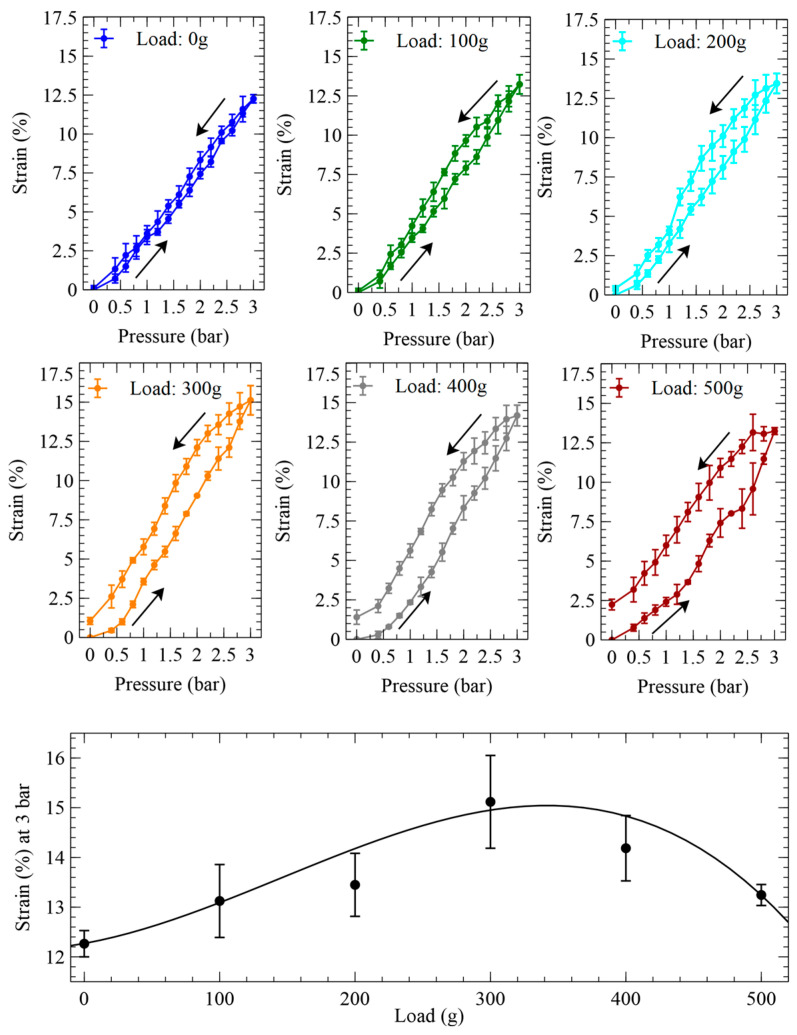
Strains generated by the inverse artificial muscle actuator in response to pressurisations and depressurisations (indicated by arrows) at different loads. The panel at the bottom plots the dependence on the load of the strain at the maximum pressure tested. The error bars represent the standard deviation among three samples. Fitting curves are used as a guide for the eye.

In order to assess the actuation performance in relative terms, each value of elongation was then used to calculate the corresponding axial strain. The length measurements and the strain calculations were performed by considering only the free length, i.e., the part of the actuator that was fully deformable, thereby excluding the portions of its extremities that were stiffened by the electrical and mechanical contacts. Figure 6 presents the pressure-induced variations of the axial strain.

The strain data show that at the maximum pressure tested (3 bars), the optimal load that maximised the actuation strain (according to the actuator’s specific size and constitutive elastomer) was 300 g, which corresponded to an axial prestress of ~150 kPa.

**Figure 7:**
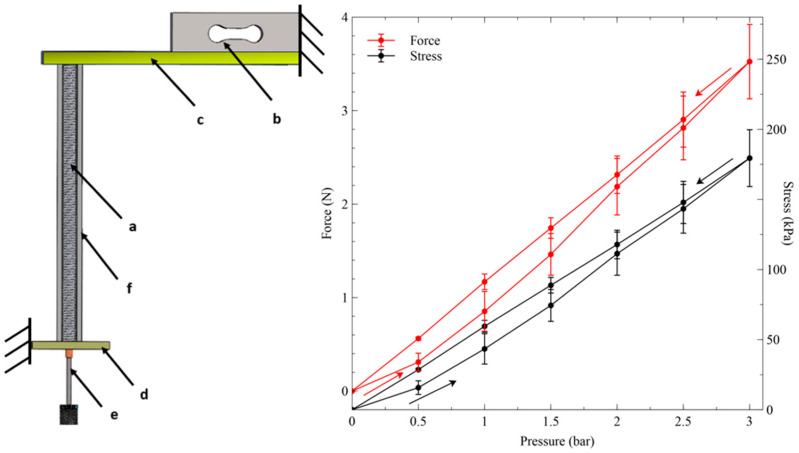
Force generated by the inverse artificial muscle actuator in response to the applied pressure at its natural length. (**Left**): drawing of the experimental setup, showing the actuator (a) in isometric condition; an axial constraint was imposed by a load cell (b) connected to a cantilever (c) and a rigid clamp at the other end (d), where the air inlet was located (e); a radial constraint was achieved with an outer stiff tube (f). (**Right**): coplots of the actuator’s axial force and stress as functions of pressurisations and depressurisations (indicated by arrows). The error bars represent the standard deviation among three samples. Linear interpolations of the data are used as a guide for the eye.

The actuation force axially produced by pressurisations and depressurisations of the device is plotted in Figure 7, which also shows the schematic of the experimental setup.

The force data show that at the maximum pressure of 3 bars, corresponding to a pressure of 300 kPa, the actuator generated a maximum axial force of ~3.5 N. In order to produce this force, the actuator did not behave just like a piston, as any pressurisation generated a total force *F* consisting of the sum of two contributions: (i) a force *F_air_* due to the direct action of the air pressure on the actuator’s internal cross-sectional area *A_air_*, i.e., the circular area identified by the tube’s inner diameter 2*r_i_* (Figure 1D), like in a piston; (ii) a force *F_tube_* exerted by the tube’s wall cross-sectional area *A_tube_* (excluding the coil’s cross section), i.e., the annular area identified by the tube’s wall thickness *r_o_−r_i_* (Figure 1D), which transduced the pressure-induced stress on the wall, from the radial to the axial direction:

(1) $$F=F_{air}+F_{tube}$$

For the pressure *P* = 300 kPa, these two contributions to the total force can easily be calculated as follows: *F_air_* = *P* × *A_air_* ≅ 2.1 N and *F_tube_* = *F − F_air_* ≅ 3.5–2.1 = 1.4 N.

The two forces corresponded to the following two contributions to the total axial stress: (i) a stress generated by the actuator’s internal cross section *σ_air_ = P*; (ii) a stress generated by the tube’s wall cross section *σ_tube_ = F_tube_*/*A_tube_*. The following linear combination of these two stresses represented the total axial stress *σ*, defined as the total force *F* divided by the total active cross-sectional area *A* = *A_air_* + *A_tube_*:

(2) $$\sigma =\frac{F_{air}+F_{tube}}{A}=\frac{{\sigma_{air}A}_{air}}{A}+\frac{{\sigma_{tube}A}_{tube}}{A}$$

Figure 7 coplots, next to the force values, the values of this total axial stress. Considering that *A_air_*/*A* = 36% and *A_tube_*/*A* = 64%, at the highest pressure *P* = 300 kPa, we had *σ_air_ = P* = 300 kPa and *σ_tube_* ≅ 110 kPa, which generated a maximum total stress *σ* ≅ 178 kPa.

**Figure 8:**
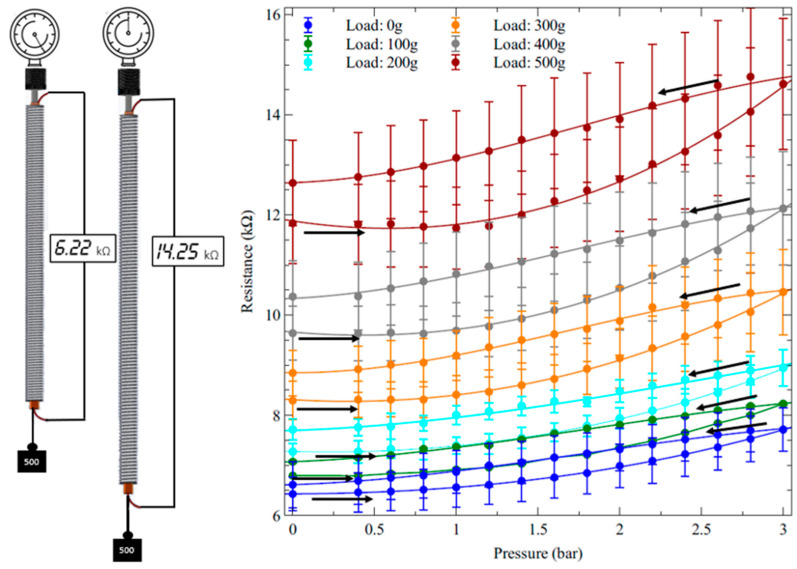
Electrical resistance of the internal piezoresistive stretch sensor of the inverse artificial muscle actuator in response to the applied pressure at different loads. (**Left**): drawing of the experimental setup, showing a pressurisation of the self-sensing actuator with an applied load. (**Right**): plots of the resistance as a function of pressurisations and depressurisations (indicated by arrows). The error bars represent the standard deviation among three samples. Fitting curves are used as a guide for the eye.

The characterisation of the sensor’s electrical resistance while the device was actuated is presented in Figure 8.

As expected, during pressurisations and depressurisations at the different loads, the resistance showed pressure-dependent variations consistent with those of the elongation.

Moreover, we can observe that the resistance (which was determined by the series between the resistance of the sensor and the resistances of its electrical contacts) had from sample to sample a certain variability: in the worst case (load of 500 g, pressure of 3 bars), the maximum uncertainty was ±1.3 kΩ for an average resistance of ~14.3 kΩ. This level of uncertainty (which was consistent with the errors on the resistance of the sensing stripe tested outside the actuator—Figure 4) was due to the variability in the manual manufacturing process of the sensor, including its electrical contacts.

**Figure 9:**
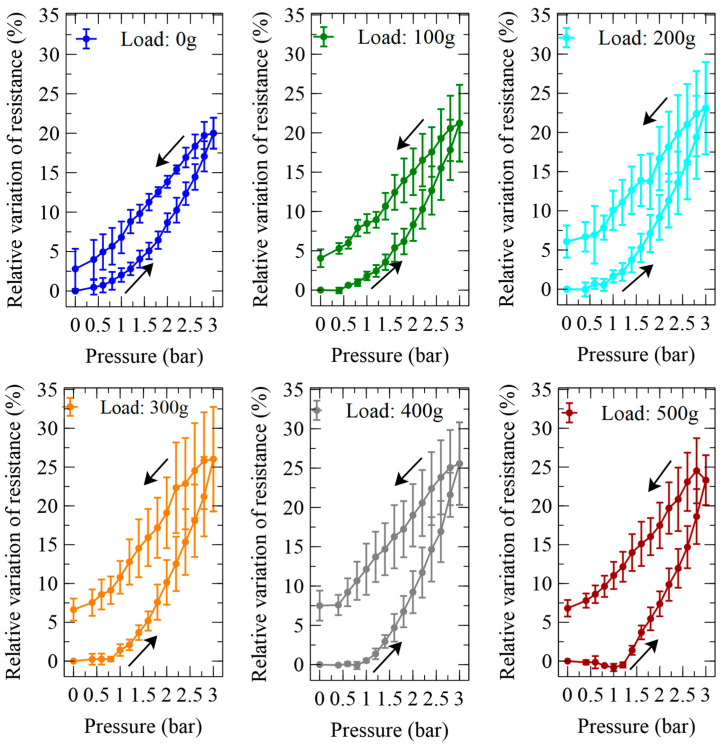
Relative variations of the electrical resistance of the piezoresistive stretch sensor internal to the inverse artificial muscle actuator, in response to pressurisations and depressurisations (indicated by arrows) at different loads. The error bars represent the standard deviation among three samples. Fitting curves are used as a guide for the eye.

In order to assess the sensing performance in relative terms, the absolute resistance data from Figure 8 were processed to obtain the pressure-induced relative variations of the electrical resistance, as presented in Figure 9.

It is worth noting that the uncertainties on the data in Figure 8 and Figure 9 are different, because Figure 8 refers to absolute measurements, whilst Figure 9 refers to relative quantifications; for each data set, the errors were calculated as the standard deviation for that data set; hence, they are different.

**Figure 10:**
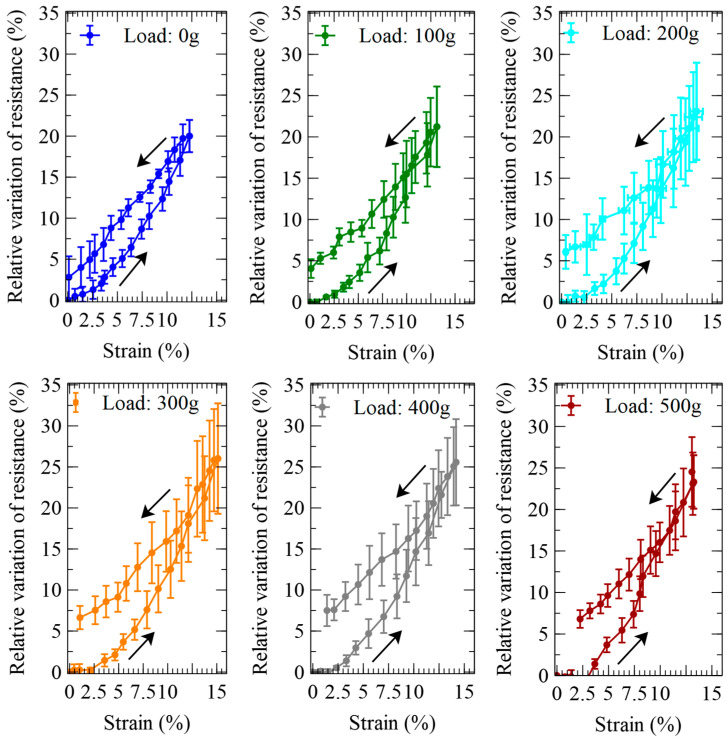
Self-sensing performance of the inverse artificial muscle actuator at different loads: relative variations of the resistance of the internal piezoresistive stretch sensor, as functions of the active strains due to pressurisations and depressurisations (indicated by arrows). The error bars represent the standard deviation among three samples. Fitting curves are used as a guide for the eye.

The self-sensing performance of the pneumatic actuator can be appreciated from Figure 10, which was obtained from a combination of the previous data sets to visualise the dependence of the sensor’s output on the pressure-induced strain.

These data show that the self-sensing actuator was affected by significant hysteresis. As the sensing stripe’s hysteresis was found to be very limited (Figure 4), the overall hysteresis might have been caused by the following two main factors. One was represented by the viscoelastic properties of the elastomeric tube, and another one by mechanical losses possibly due to structural effects. The latter might have originated from the Nylon coil sliding over the tube. Indeed, as the tube was pressed against the Nylon line, friction with the silicone surface caused energy dissipation, which was different during inflation (extension) and deflation (shrinkage) of the tube.

The significant hysteresis would create a problem for a reliable use of this sensing strategy, owing to the lack of a univocal curve that can link the sensor’s output and input variables (calibration curve). Indeed, any given variation of resistance cannot be associated with a single value of strain (Figure 10). This implies that in order to make the sensing strategy practically usable, it would be necessary to adopt a certain criterion that avoids the indetermination, by accepting a certain sensing error. The most straightforward approach would be to consider as a calibration curve the average curve between the two branches of the hysteretic loop. However, the large area of the loop, i.e., the large distance between the two branches, would cause a large error.

**Figure 11:**
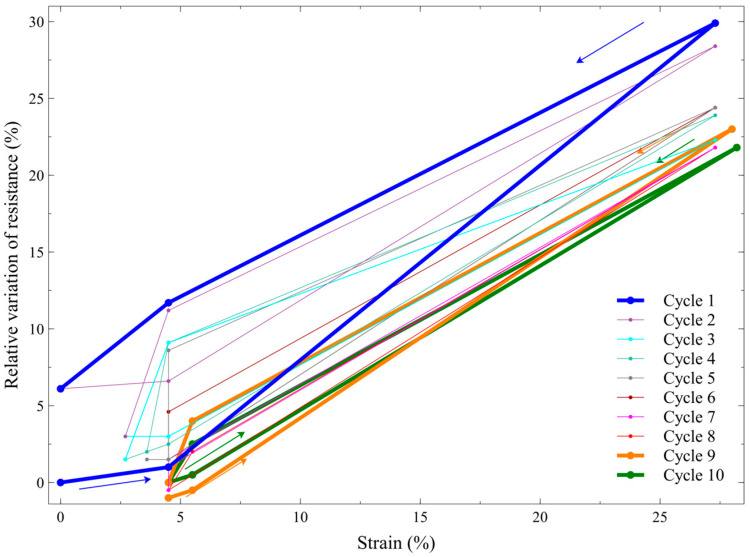
Self-sensing performance of one sample of the inverse artificial muscle actuator, during ten consecutive cycles of pressurisation up to 3 bars and depressurisation to 0 (indicated by arrows). The relative variations of resistance and strain were calculated with respect to the resistance and length values at the beginning of the 1st cycle.

We therefore further characterised the hysteretic behaviour, by investigating whether it changed with an increasing number of cycles. To this aim, one sample of the self-sensing actuator with a 300 g loading (which ensured the highest active strains—Figure 6) was pressurised up to 3 bars and depressurised to zero, a total of 10 times. Between any two consecutive cycles, the actuator was maintained depressurised for 10 min, so as to facilitate recovery from creep. The results are presented in Figure 11.

These data show that, after the first cycle, the hysteretic loop progressively decreased its area. Moreover, for each cycle, the initial strain tended to slightly increase with respect to the preceding cycle; this means that the actuator progressively accumulated a residual strain, which reached a value of ~4.5% at the 10th cycle (Figure 11). Therefore, the recovery from creep between two consecutive cycles was incomplete. Nevertheless, more importantly, these data show that after the ninth cycle there was no further shift, i.e., the hysteretic loop stabilised its position. Moreover, the loop’s area became so small that it enabled an accurate use of a linear calibration, i.e., a linearisation of the sensor’s response. Indeed, by defining the calibration relationship as the line passing through the extreme points of the 10th cycle’s loop, the maximum sensing error (numerically determined from Figure 11) within the experimented range of strains (up to nearly 30%) was found to be a strain of ±1.6%.

This evidence indicates that, after an initial preconditioning phase, which is required to stabilise the response, the proposed self-sensing strategy may effectively be used to continuously monitor the artificial muscle actuator’s strain during operation with adequate sensing accuracy.

Nevertheless, future systematic studies are necessary to investigate whether the achieved stabilisation of the hysteretic behaviour would be retained over time and over repeated cycling. Especially, extensive cyclic tests should be conducted to assess how both the actuation and the self-sensing responses may be affected by fatigue.

Another issue with the sensor is represented by the fact that, to date, we do not know whether its location inside the tube may lead or not to undesired effects. For instance, future investigations should explore whether (and, if so, under what circumstances) pressurisations could induce in the suspended sensor unexpected asymmetric deformations, or even vibrations, such that the sensing signal might lead to misleading interpretations. If so, the problem should be studied in detail, in order to devise possible solutions. For instance, suitable strategies might involve not only signal filtering, but also possible combinations of multiple sensing elements for accurate identifications and corrections.

**Figure 12:**
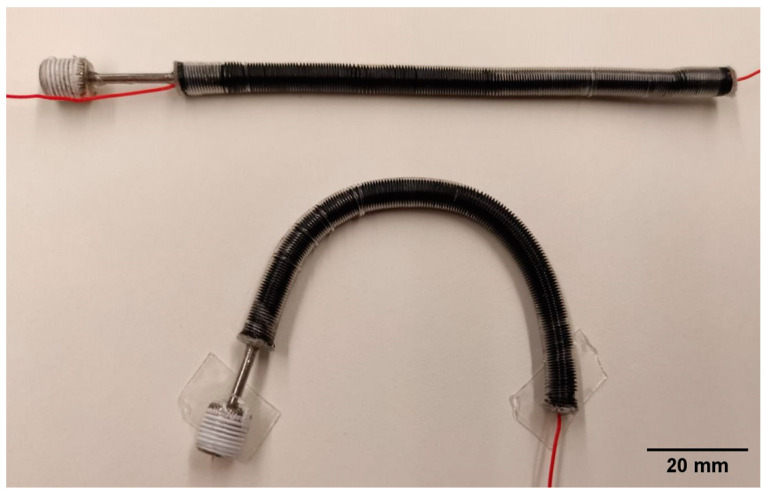
Possible alternative solution to obtain a self-sensing inverse artificial muscle actuator, without affecting the constraining coil and without adding any internal sensor: the elastomeric tube is made of a carbon-loaded silicone, so that it can serve not only as the pressurisation chamber, but also as a piezoresistive stretch sensor.

On the other hand, the location of the sensor might be changed. Whilst in this study it was arranged coaxially with the actuator (with the intended advantages described in the Introduction), in [33] it was inserted inside the constraining coil, which was made soft on purpose. Different strategies might also be possible. For instance, the elastomeric tube itself could be made intrinsically conductive, so as to use it both as the pressurisation chamber and as a piezoresistive stretch sensor. We are currently exploring such an alternative strategy, by testing actuators made of a carbon-loaded silicone tube, as shown in Figure 12.

Yet another alternative may be to provide the elastomeric tube with the ability to work also as a piezocapacitive stretch sensor. To this end, both its inner and outer surfaces should be coated with a thin layer of a conductive elastomer (e.g., a carbon-loaded silicone), so as to create two compliant electrodes. The resulting tube sandwiched between the two electrodes would therefore act as a deformable capacitor, able to sense its own strain by means of a variation of electrical capacitance.

## 4. Conclusions

This paper presented the architecture, a manufacturing process and a preliminary characterisation of a new version of self-sensing pneumatic actuators, serving as inverse artificial muscles. The actuators were made with off-the-shelf materials and a simple fabrication process, which was detailed with video clips.

The proposed self-sensing strategy is simple and scalable. Moreover, it advantageously does not require any modification to the radially constraining coil. This allows the coil to be made with flexible (and not stretchable) materials, thereby preserving its optimal mechanical function.

## Data Availability

The data presented in this study are available on request from the corresponding authors.

## Supplementary Materials

The following supporting information can be downloaded at: https://www.mdpi.com/article/10.3390/biomimetics9030177/s1, Video clips ‘Fabrication step 1’, ‘Fabrication step 2’, ‘Fabrication step 3’ and ‘Fabrication step 4’.
